# Editorial: Diabetes complications in children and adolescents: from low-resource to technology-advanced countries

**DOI:** 10.3389/fmed.2025.1567692

**Published:** 2025-02-20

**Authors:** Giulio Frontino, Martina Matarazzo, Roberto Franceschi, Enza Mozzillo, Marco Marigliano

**Affiliations:** ^1^Department of Pediatrics, IRCCS San Raffaele Hospital, Milan, Italy; ^2^Diabetes Research Institute, IRCCS San Raffaele Hospital, Milan, Italy; ^3^Centre for Medical Sciences (CISMed), Department of Cellular, Computational and Integrative Biology (CIBIO), University of Trento, Trento, Italy; ^4^Department of Translational Medical Science, Section of Pediatrics, Università degli Studi di Napoli Federico II, Naples, Italy; ^5^Department of Surgery, Dentistry, Pediatrics and Gynecology, Section of Pediatric Diabetes and Metabolism, University and Azienda Ospedaliera Universitaria Integrata of Verona, Verona, Italy

**Keywords:** type 1 diabetes (T1D), diabetes technology, advanced hybrid closed-loop, diabetes complications, diabetic neuropathy (DN)

Pediatric diabetes is a global health concern with significant inequalities and unmet needs in healthcare infrastructure, technological access, and funding (1). Diabetic ketoacidosis (DKA), neuropathy, and severe hypoglycemia are diabetes-related complications that place a significant cost and burden on patients, families, and healthcare systems (2–4). The worldwide rising incidence of type 1 diabetes (T1D) makes further deteriorates this situation (5). Therefore, creating solutions that address acute and long-term complications in various contexts is crucial. People with access to advanced technology have trouble incorporating state-of-the-art technologies into routine care (6). Those in resource-limited nations struggle with basic diabetic management. This Research Topic aims to bridges the gap between resource inequities and innovative solutions in the context of diabetes complications in children.

The study by Lazar et al. proposes anion gap normalization time (AGNT) as a more reliable marker for diabetic ketoacidosis (DKA) resolution in pediatric patients, challenging traditional reliance on pH and bicarbonate levels. Their findings indicate that AG normalizes significantly earlier than these markers, with a median AGNT of 8 h, preceding PICU discharge by 15 h. Notably, severe acidosis (pH < 7.1) and hyperglycemia (>500 mg/dL) were strong predictors of delayed AGNT, reinforcing its potential as a clinically relevant indicator. By adopting AGNT as a criterion for de-escalation of intensive care, clinicians may reduce unnecessary PICU stays, minimize patient burden, and optimize hospital resource utilization. However, the study's retrospective nature and absence of ketone body measurements necessitate further prospective validation before AGNT can be fully integrated into DKA management protocols. This research raises an important question: should anion gap normalization replace conventional DKA resolution markers, paving the way for a more efficient and patient-centered approach? (Lazar et al.).

The study by Tinti et al. explores the long-term neurological dysfunction in adolescents with type 1 diabetes, highlighting early subclinical alterations in nerve conduction. Using nerve conduction studies (NCS) and follow-up neuropathy screening questionnaires, the study found that while 11.8% of patients showed signs of somatic dysfunction, a significant 41% had reduced hypoglycemia awareness, suggesting early autonomic impairment. Importantly, an abnormal NCS outcome correlated with a 97-fold increased risk of severe hypoglycemia after 6 years, emphasizing the potential impact of diabetic autonomic neuropathy (DAN) on metabolic control. Despite the small sample size and lack of comprehensive metabolic data, the findings reinforce the need for early screening for neuropathy in pediatric diabetes, particularly through electrophysiological assessments that may identify risk factors before clinical symptoms appear. This study aligns with growing evidence that early autonomic dysfunction in diabetes can lead to increased glycemic variability and hypoglycemia risk, advocating for a more proactive approach in pediatric neuropathy screening to improve long-term diabetes management (Tinti et al.).

The study by Leutheuser et al. investigates the prediction of nocturnal hypoglycemia in children with type 1 diabetes following daytime physical activity using machine learning models applied to continuous glucose monitoring (CGM) and physiological data. The study, conducted in a structured sports camp setting, assessed logistic regression, random forest, and deep neural networks to forecast hypoglycemia over a 9-h overnight period. Results showed that random forest models using only glucose data achieved the highest predictive accuracy (F2 score: 64.4%), while incorporating physiological data marginally improved performance. Despite variability in model effectiveness, the findings underscore the clinical importance of early hypoglycemia risk prediction, allowing caregivers to take proactive measures, such as adjusting insulin or carbohydrate intake, to prevent overnight complications. However, the small sample size (13 children, 66 nights) and dataset constraints highlight the need for larger-scale validation studies. This research contributes to the growing field of AI-driven diabetes management, but further refinements, including personalized modeling and real-world validation, are essential before clinical implementation (Leutheuser et al.).

The study by López-López et al. evaluates the real-world efficacy and safety of the MiniMed 780G advanced hybrid closed-loop (AHCL) system in children under 7 years of age with type 1 diabetes (T1D). Analyzing data from 61 pediatric patients across three endocrinology centers in the Canary Islands, the study found that time in range (TIR) increased significantly (up to 13%−15%), while time above range (TAR) decreased without an increase in hypoglycemia risk. These metabolic improvements were sustained for up to 12 months, supporting the long-term benefits of AHCL therapy in young children. Importantly, the study also assessed children requiring low total daily insulin doses (< 8 units/day) and found no adverse impact on safety or efficacy, suggesting that AHCL systems may be suitable even for those with minimal insulin needs. Despite the retrospective design and small sample size, the findings reinforce the role of automated insulin delivery in achieving optimal glycemic control in very young children with T1D, advocating for its expanded use and further validation in larger cohorts (López-López et al.).

## Implications for global diabetes care

These studies highlight the need for adaptable solutions to address disparities in diabetes care across different healthcare systems. Complex technologies may not be feasible in low-resource settings, but simple and effective tools like anion gap normalization time (AGNT) and scalable neuropathy screening methods can significantly improve outcomes. These approaches enable early intervention and better complication management without requiring high-cost infrastructure. Cost-effective, evidence-based strategies tailored to resource-limited environments will be key to ensuring equitable access to quality diabetes care (7).

Advancements in machine learning (ML)-based predictions and advanced hybrid closed-loop (AHCL) systems have transformed diabetes management in technologically developed settings. These innovations help optimize glycemic control, reduce fluctuations, and lower the risk of complications, enhancing the overall quality of life for people with type 1 diabetes (8). However, these technologies' high cost and limited availability create barriers for many individuals, especially in low- and middle-income countries. Bridging this gap requires collaboration among healthcare providers, policymakers, and industry leaders to develop policies that ensure these life-changing innovations become affordable and widely accessible.

Equitable diabetes care depends on global cooperation. High-resource institutions should take an active role in capacity-building efforts by investing in training programs, telemedicine support, and knowledge-sharing initiatives for healthcare providers in under-resourced regions. Strengthening local expertise and improving access to essential technologies, such as continuous glucose monitoring (CGM) and AHCL systems, can empower clinicians to provide better care for children with type 1 diabetes. Policymakers must prioritize investment in diabetes-related healthcare policies, ensuring that affordability, and accessibility remain at the forefront of decision-making.

Future research must also consider socioeconomic, cultural, and regional differences to create scalable and practical solutions. Interventions should be designed to meet the specific needs of diverse populations, accounting for variations in healthcare infrastructure, patient education, and treatment accessibility. Sustainable progress in diabetes management will rely on balancing technological innovation with real-world feasibility, ensuring that all children, regardless of their location, or financial background, receive the care they need to thrive.

## Conclusions

This Research Topic highlights how innovation and research help manage diabetes complications in children and adolescents. By reducing resource gaps and using technology, the global diabetes community can improve care for all young patients. Achieving this goal requires teamwork, advocacy, and a strong commitment to health equity.
